# Arthropod-Borne Disease Control at a Glance: What’s New on Drug Development?

**DOI:** 10.3390/molecules25215175

**Published:** 2020-11-06

**Authors:** Giovanni Benelli, Riccardo Petrelli, Angelo Canale

**Affiliations:** 1 Department of Agriculture, Food and Environment, University of Pisa, via del Borghetto 80, 56124 Pisa, Italy; angelo.canale@unipi.it; 2 School of Pharmacy, University of Camerino, 62032 Camerino, Italy; riccardo.petrelli@unicam.it

**Keywords:** Chagas disease, chikungunya, dengue, human African trypanosomiasis, Japanese encephalitis, malaria, leishmaniasis, lymphatic filariasis, West Nile fever, yellow fever, Zika virus

## Abstract

Discovering and validating effective drugs to manage arthropod-borne diseases (ABD) is a timely and important research challenge with major impacts on real-world control programs at the time of quick resistance development in the targeted pathogens. This editorial highlights major research advances in the development of drugs for the control of vector-borne diseases, with a significant focus on malaria, Chagas disease, dengue, human African trypanosomiasis, leishmaniasis, and Zika. Broad reviews providing new insights on ABD recently published in *Molecules* have also been covered in “The Editors’ pick” section.

## 1. Introduction

Vector-borne diseases (VBD) lead to more than 700,000 deaths yearly, with malaria and dengue alone causing >400,000 and 40,000 deaths per year, respectively [1]. A relevant number of VBD are spread by arthropod vectors. Mosquitoes, sandflies, blackflies, tsetse flies, kissing bugs, and ticks, among others, are key examples, with huge public health impacts. Their control is a major goal for public health. Simultaneously, developing drugs to manage arthropod-borne diseases (ABD) is a timely and important research challenge with a major impact on real-world ABD elimination/control programs [2,3,4]. The current scenario is worsened by the rapid increase in drug resistance in targeted pathogens [5,6,7], coupled with the dangerous spread of invasive arthropod vectors (e.g., the Asian tiger mosquito, *Aedes albopictus*, and the cattle tick, *Rhipicephalus* (*Boophilus*) *microplus*) [8,9], facilitated by anthropic activities, with special reference to trades, urbanization, and global warming [10,11,12,13].

Performing excellently from a scientometric point of view, *Molecules* currently represents a first-class journal for publishing researches and reviews on the development of novel and effective drugs to manage ABD. In this framework, several Special Issues covering this topic are ongoing, ensuring higher visibility for the authors compared to regular issues. As Academic Editors, we invite all readers to submit their forthcoming original research and reviews on ABD drug development to *Molecules*.

## 2. The Editors’ Pick

In this section, a carefully reviewed selection of cutting edge articles published in *Molecules* during the period 2019-2020 is highlighted. To our eyes, these studies represent key research advances regarding the development of drugs for the control of ABD. Furthermore, recent reviews providing well-updated scenarios about drug development against selected ABD and formulating new research challenges are outlined [14,15,16,17,18,19,20,21,22,23]. Among key ABD, major focuses have been malaria, Chagas disease, dengue, human African trypanosomiasis (HAT), leishmaniasis, and Zika.

Cutting edge articles about drugs to manage arthropod-borne diseases recently published in *Molecules*:

**Lapatinib, Nilotinib and Lomitapide Inhibit Haemozoin Formation in Malaria Parasites**
by Ana Carolina C. de Sousa, Keletso Maepa, Jill M. Combrinck and Timothy J. Egan
*Molecules***2020**, 25(7), 1571; https://doi.org/10.3390/molecules25071571

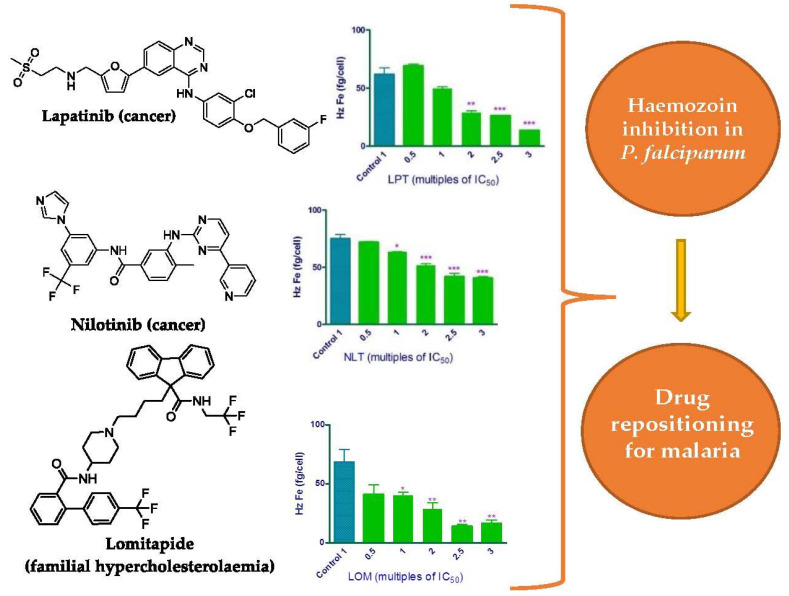

**Fungal Metabolite Asperaculane B Inhibits Malaria Infection and Transmission**
by Guodong Niu, Yue Hao, Xiaohong Wang, Jin-Ming Gao and Jun Li
*Molecules***2020**, 25(13), 3018; https://doi.org/10.3390/molecules25133018

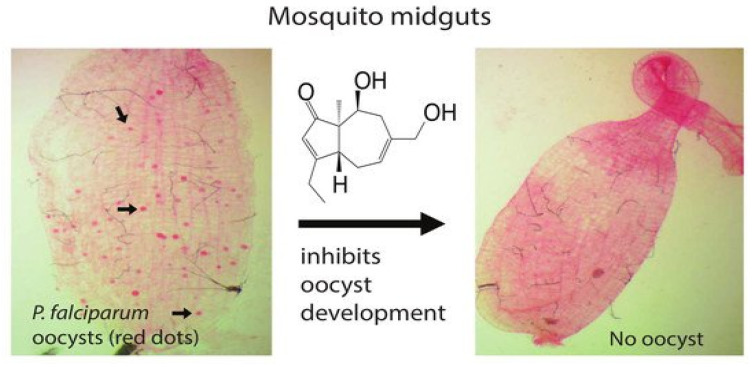

**Alkyl and Aryl Derivatives Based on *p*-Coumaric Acid Modification and Inhibitory Action against *Leishmania braziliensis* and *Plasmodium falciparum***
by Susiany P. Lopes, Lina M. Yepes, Yunierkis Pérez-Castillo, Sara M. Robledo and Damião P. de Sousa
*Molecules***2020**, *25*(14), 3178; https://doi.org/10.3390/molecules25143178

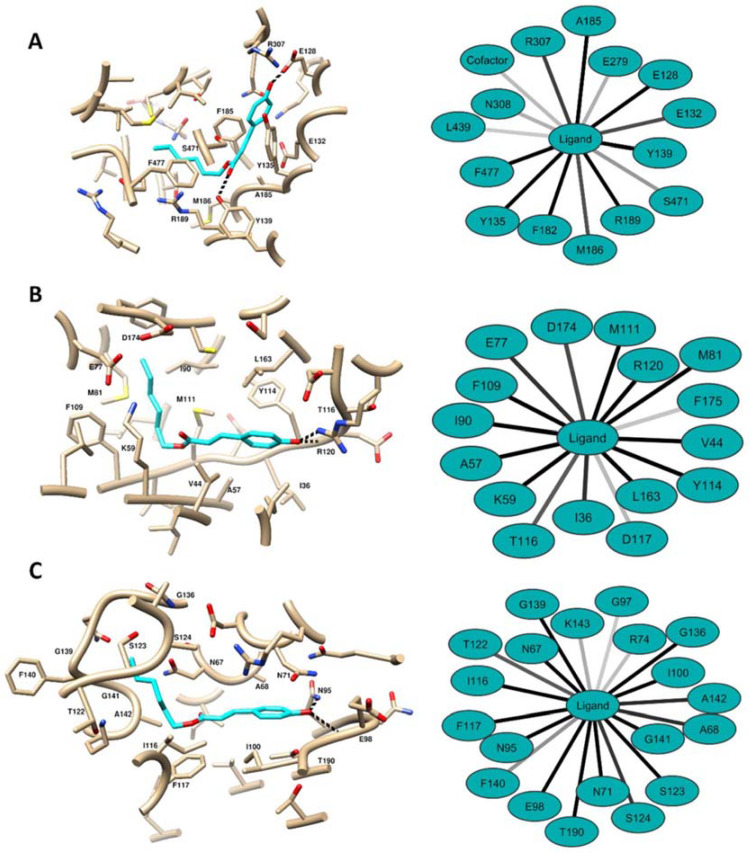

**4-Arylthieno[2,3-*b*]pyridine-2-carboxamides Are a New Class of Antiplasmodial Agents**
by Sandra I. Schweda, Arne Alder, Tim Gilberger and Conrad Kunick
*Molecules***2020**, 25(14), 3187; https://doi.org/10.3390/molecules25143187

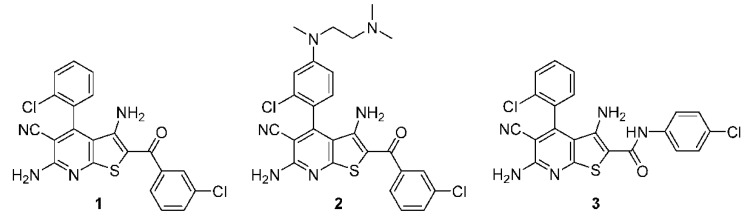

**Cytotoxic and Anti-Plasmodial Activities of *Stephania dielsiana* Y.C. Wu Extracts and the Isolated Compounds**
by James Knockleby, Bruno Pradines, Mathieu Gendrot, Joel Mosnier, Thanh Tam Nguyen, Thi Thuy Trinh, Hoyun Lee, and Phuong Mai Le
*Molecules***2020**, 25(16), 3755; https://doi.org/10.3390/molecules25163755

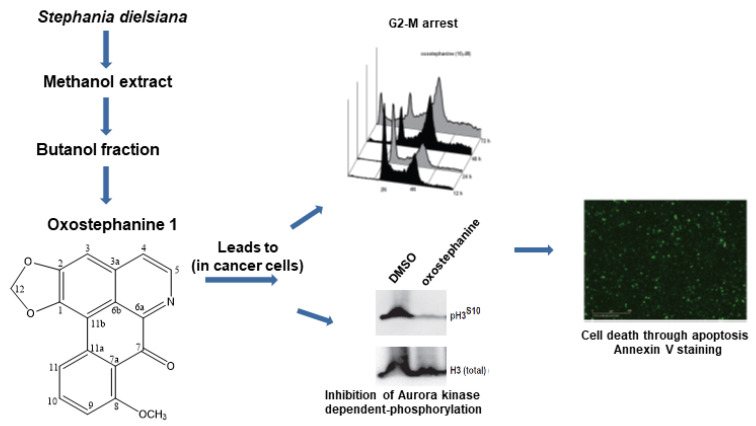

**Andrographolide and Its 14-Aryloxy Analogues Inhibit Zika and Dengue Virus Infection**
by Feng Li, Wipaporn Khanom, Xia Sun, Atchara Paemanee, Sittiruk Roytrakul, Decai Wang, Duncan R. Smith and Guo-Chun Zhou
*Molecules***2020**, 25(21), 5037; https://doi.org/10.3390/molecules25215037

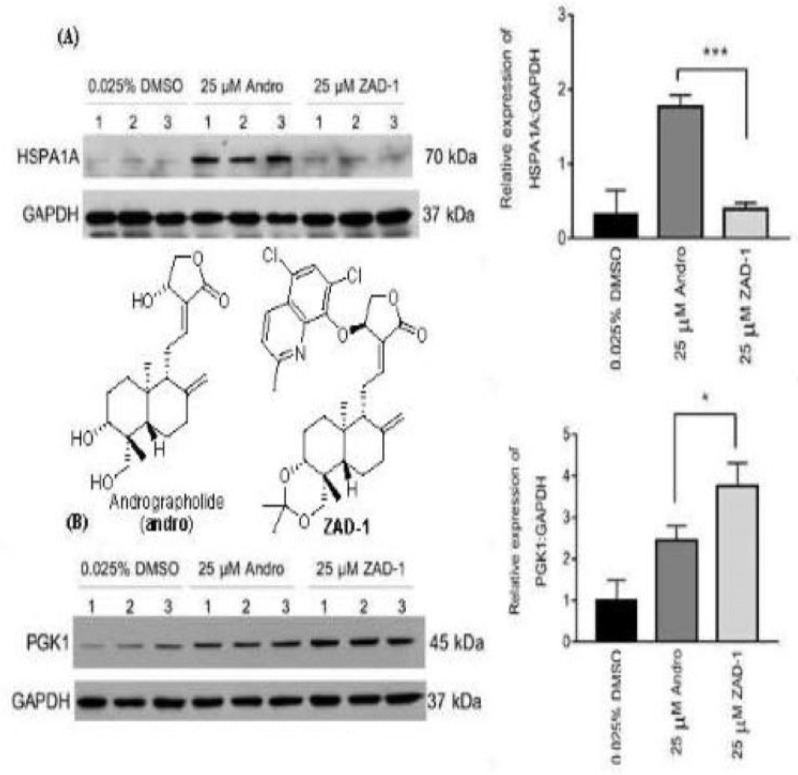

**Harringtonine Inhibits Zika Virus Infection through Multiple Mechanisms**
by Zheng-Zong Lai, Yi-Jung Ho and Jeng-Wei Lu
*Molecules***2020**, 25(18), 4082; https://doi.org/10.3390/molecules25184082

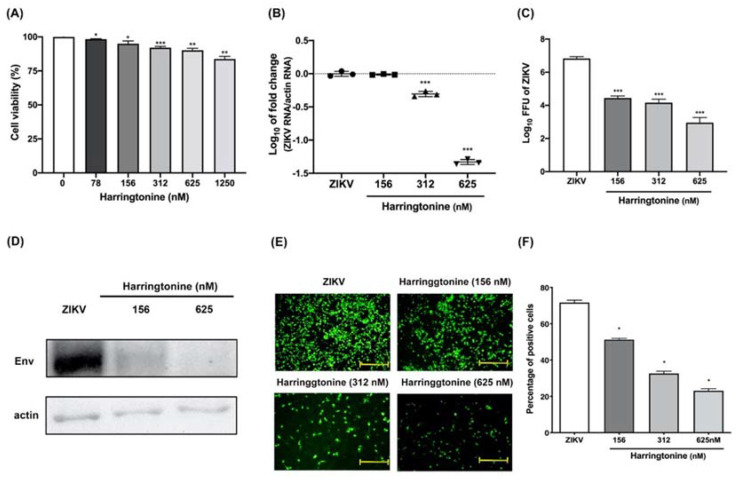

**In Vitro and In Vivo Effectiveness of Carvacrol, Thymol and Linalool against *Leishmania infantum***
by Mohammad Reza Youssefi, Elham Moghaddas, Mohaddeseh Abouhosseini Tabari, Ali Akbar Moghadamnia, Seyed Mohammad Hosseini, Bibi Razieh Hosseini Farash, Mohammad Amin Ebrahimi, Niki Nabavi Mousavi, Abdolmajid Fata, Filippo Maggi, Riccardo Petrelli, Stefano Dall’Acqua, Giovanni Benelli and Stefania Sut
*Molecules***2019**, 24(11), 2072; https://doi.org/10.3390/molecules24112072

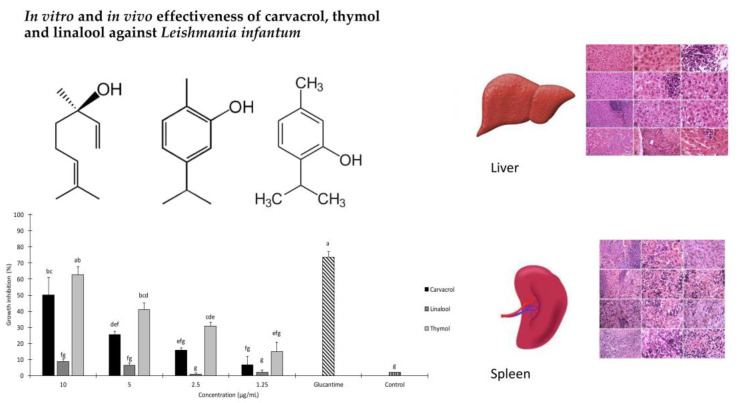

**Trypanocidal Essential Oils: A Review**
by Mayara Castro de Morais, Jucieudo Virgulino de Souza, Carlos da Silva Maia Bezerra Filho, Silvio Santana Dolabella and Damião Pergentino de Sousa
*Molecules***2020**, *25*(19), 4568; https://doi.org/10.3390/molecules25194568

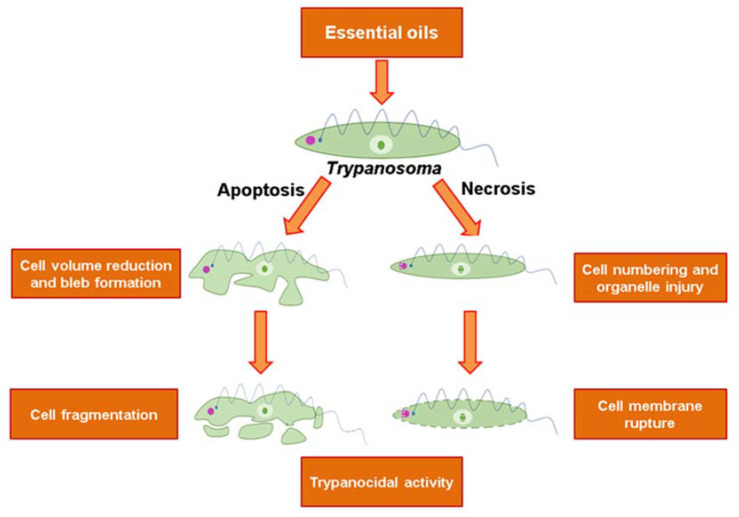

**Antiviral Natural Products for Arbovirus Infections**
by Vanessa Shi Li Goh, Chee-Keng Mok and Justin Jang Hann Chu
*Molecules***2020**, 25(12), 2796; https://doi.org/10.3390/molecules25122796

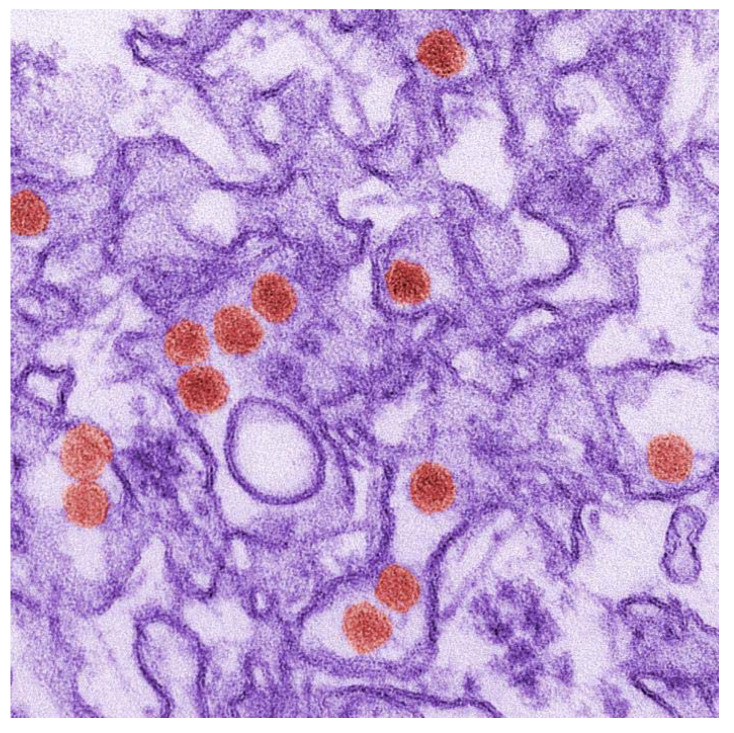


## 3. Conclusions and Challenges

Overall, the development of novel and effective products to control ABD is receiving major research attentions and efforts. *Molecules* is strongly supportive of this research field, with a special focus on emerging ABD, as shown in the Zika virus example. Furthermore, much remains to be done in managing ABD of “historical” public health importance, such as malaria, which is still threatening a major number of countries worldwide. In this scenario, natural products represent a huge reservoir of bioactive substances of potential interest for drug development, as well as for designing insecticides, acaricides, and repellents for vector management actions [24,25,26,27]. Of note, in many cases a relevant link between the ethnobotanical report and the scientific evidence proving its efficacy has been highlighted.
